# Supplementary material for: Development of a Clinical Prediction Rule for Treatment Success with Transcranial Direct Current Stimulation for Knee Osteoarthritis Pain: A Secondary Analysis of a Double-Blind Randomized Controlled Trial
Source: Biomedicines. 2022 Dec 20;11(1):4. doi: 10.3390/biomedicines11010004 (PMC9855334; doi:10.3390/biomedicines11010004)
Supplement: Supplementary file 1 [file biomedicines-11-00004-s001.zip › biomedicines-2006094-supplementary.pdf]

Summary of the main RCT study procedures:

Participants

Participants were recruited from São Paulo hospital outpatient clinics and the general population using online and local newspaper advertising, social media, and community flyers. Patients were eligible if (1) aged 60 years or older, (2) had diagnosis of KOA according to the American College of Rheumatology criteria, (3) with KOA pain for at least the past six months, with (4) a pain intensity average rated as "4" or more on a 0-10 numerical rating scale, and (5) with a dysfunctional DPIS, defined according to Tarrago et al[1]. In summary, patients were excluded if they have had history of substance abuse, seizures or loss of consciousness, severe depression and/ or anxiety, any diagnosed neurological conditions, or contraindications to tDCS. Details of inclusion and exclusion criteria were reported previously[2]. The fifty-one subjects who were randomly allocated to the active tDCS group were used for this study and baseline demographics are reported elsewhere[3].

Intervention

Participants received 15 daily sessions of stimulations performed during weekdays (Monday to Friday) for three weeks. tDCS stimulations were performed using the Soterix (Medical 1X1 Low-Intensity Stimulator, Soterix Medical Inc), device via two 35 cm^2 surface sponge electrodes (EASYpad™ Soterix Medical Inc.) soaked with saline solution. The device parameters included a current intensity of 2 mA that was set to gradually ramp up and down for the first and last 30 seconds of the application, respectively. The anode electrode was placed over the primary motor cortex (M1) area (C3/C4 placement

according to the 10-20 electroencephalogram system) contralateral to the most symptomatic knee. The cathode electrode was placed at the supraorbital area contralateral to the anode. The stimulation had a duration of 20 minutes per session, and it was performed by the same physician who was experienced and trained on the stimulation technique. All stimulations happened at the same period (morning) of the day for all patients.

A detailed explanation of the outcome assessments used in the main clinical trial is reported elsewhere[2,3]. However, a brief description of the clinical variables is listed below:

Pain Related Variables

The Brief Pain Inventory scores included both the pain interference and the pain intensity dimension sub-scores. While the pain interference score assessed how much pain interferes in functional activities, the pain intensity dimension considers the pain intensity at the moment of assessment, in addition to the average, the worst, and the least level of pain on the last 24 hours using a numeric rating scale of 0-10 (no pain – extreme pain)[4,5]. Additionally, the visual analog scale for pain was also used for the pain at the moment of assessment and over the past seven days[6]. For both assessment tools, higher scores represent higher levels of pain and/or pain interference.

Psychosocial Related Variables

The Visual Analog Mood Scale (VAMS)[7,8] was used as a measure of anxiety, depression, stress, and sleepiness[9]. Mood was reported only for the moment of the

assessment. The Beck Depression Inventory was used to evaluate depression severity symptoms[10]. For both measurement tools, higher scores indicate higher mood intensity or depression symptoms.

Self-Reported Health-Related Quality of Life

Quality of life scores from the mental (MCS) and physical (PCS) components of the 12-item Short Form Health Survey questionnaire were used to assess quality of life. Subscale scores range from 0-100 where lower the scores, poorer quality of life [11]. Subjects were also asked to self-rate their general health using a visual analog scale.

Disease Specific Measures

The Western Ontario and McMaster Universities Osteoarthritis Index (WOMAC) and the Lequesne Algofunctional Index were used as disease specific, health status measures. The WOMAC's three dimension scores (pain, stiffness, and physical function), in addition to its total score were considered as exploratory variables[12,13]. The Lequesne Algofunctional Index[14,15] subscales (pain or discomfort, maximum distance walked, activities of daily living) were also considered separately as well as its total score. For both tools, higher scores represent higher levels of disability/pain/symptoms.

Performance-Based Physical Function

The timed up-and-go test (TUGT)[16,17] and the one-leg stance test[18] were used as performance-based functional measures. The time it took to perform the task was

recorded in seconds for the TUGT, while the time spent balancing in one leg was used for the leg stance test.

Quantitative Sensory Testing Variables

Mechanical detection and mechanical pain thresholds were measured using the Von Frey monofilament[19] (Von Frey monofilament, Touch-Test Sensory Evaluator, North Coast Medical, Morgan Hill, CA, USA) as described previously[2,9]. Single applications of standardized monofilaments were applied first to a reference region (thenar region, ipsilateral to the side of most painful knee), followed by applications to a knee region (the most painful knee)[19,20].

Pain Pressure Threshold

Pain pressure threshold (PPT) was assessed by means of blunt pressure delivered by a 1-cm^2 hard-rubber algometer probe (Commander Echo Algometer, JETCH)[21]. Three discrete pressures were applied at both the reference and knee locations and scores were recorded in kilograms per cm^2, and averaged. PPT was defined and recorded as the amount of pressure required to elicit a pain sensation distinct from pressure or discomfort [9].

Conditioned Pain Modulation

The Conditioned Pain Modulation (CPM) technique was performed as described elsewhere[2]. CPM was defined as the percentage change in pain scores and PPT after CPM procedure. Both of them were calculated according to the equation: (post-CPM trial

score - pre-CPM trial score/pre-CPM trial score) x 100. Negative values for the percentage change in pain scores after CPM indicated a functional pain inhibition, while positive values for the change in PPT after CPM.

REFERENCES

1. Tarrago Mda G, Deitos A, Brietzke AP, et al. Descending Control of Nociceptive Processing in Knee Osteoarthritis Is Associated With Intracortical Disinhibition: An Exploratory Study. *Medicine*. 2016;95(17):e3353. doi:10.1097/md.0000000000003353

2. Tavares DRB, Okazaki JEF, Rocha AP, et al. Effects of Transcranial Direct Current Stimulation on Knee Osteoarthritis Pain in Elderly Subjects With Defective Endogenous Pain-Inhibitory Systems: Protocol for a Randomized Controlled Trial. *JMIR Res Protoc*. 2018;7(10):e11660. doi:10.2196/11660

3. Tavares DRB, Okazaki JEF, Santana MV de A, et al. Motor cortex transcranial direct current stimulation effects on knee osteoarthritis pain in elderly subjects with dysfunctional descending pain inhibitory system: A randomized controlled trial. *Brain Stimul*. 2021;14(3):477-487. doi:10.1016/j.brs.2021.02.018

4. Kapstad H, Rokne B, Stavem K. Psychometric properties of the Brief Pain Inventory among patients with osteoarthritis undergoing total hip replacement surgery. *Health Qual Life Outcomes*. 2010;8:148. doi:10.1186/1477-7525-8-148

5. Kapstad H, Hanestad BR, Langeland N, Rustøen T, Stavem K. Cutpoints for mild, moderate and severe pain in patients with osteoarthritis of the hip or knee ready for joint replacement surgery. *BMC Musculoskelet Disord*. 2008;9:55. doi:10.1186/1471-2474-9-55

6. Kersten P, White PJ, Tennant A. Is the pain visual analogue scale linear and responsive to change? An exploration using Rasch analysis. *PLoS ONE*. 2014;9(6):e99485. doi:10.1371/journal.pone.0099485

7. Ahearn EP. The use of visual analog scales in mood disorders: a critical review. *J Psychiatr Res*. 1997;31(5):569-579.

8. Cella DF, Perry SW. Reliability and Concurrent Validity of Three Visual-Analogue Mood Scales. *Psychol Rep*. 1986;59(2):827-833. doi:10.2466/pr0.1986.59.2.827

9. Teixeira PEP, Zehry HI, Chaudhari S, Dipietro L, Fregni F. Pain perception in chronic knee osteoarthritis with varying levels of pain inhibitory control: an exploratory study. *Scand J Pain*. 2020;20(4):651-661. doi:10.1515/sjpain-2020-0016

10. Warmenhoven F, van Rijswijk E, Engels Y, et al. The Beck Depression Inventory (BDI-II) and a single screening question as screening tools for depressive disorder in Dutch advanced cancer patients. *Support Care Cancer*. 2012;20(2):319-324. doi:10.1007/s00520-010-1082-8

11. Webster KE, Feller JA. Comparison of the short form-12 (SF-12) health status questionnaire with the SF-36 in patients with knee osteoarthritis who have replacement surgery. *Knee Surg Sports Traumatol Arthrosc*. 2016;24(8):2620-2626. doi:10.1007/s00167-015-3904-1

12. Basaran S, Guzel R, Seydaoglu G, Guler-Uysal F. Validity, reliability, and comparison of the WOMAC osteoarthritis index and Lequesne algofunctional index in Turkish patients with hip or knee osteoarthritis. *Clin Rheumatol*. 2010;29(7):749-756. doi:10.1007/s10067-010-1398-2

13. Bruce B, Fries J. Longitudinal comparison of the Health Assessment Questionnaire (HAQ) and the Western Ontario and McMaster Universities Osteoarthritis Index (WOMAC). *Arthritis Care & Research*. 2004;51(5):730-737. doi:10.1002/art.20695

14. Faucher M, Poiraudeau S, Lefevre-Colau MM, Rannou F, Fermanian J, Revel M. Assessment of the test-retest reliability and construct validity of a modified Lequesne index in knee osteoarthritis. *Joint Bone Spine*. 2003;70(6):521-525. doi:10.1016/s1297-319x(03)00070-8

15. Faucher M, Poiraudeau S, Lefevre-Colau MM, Rannou F, Fermanian J, Revel M. Algo-functional assessment of knee osteoarthritis: comparison of the test-retest reliability and construct validity of the WOMAC and Lequesne indexes. *Osteoarthritis Cartilage*. 2002;10(8):602-610. doi:10.1053/joca.2002.0533

16. Sabirli F, Paker N, Bugdayci D. The relationship between Knee Injury and Osteoarthritis Outcome Score (KOOS) and Timed Up and Go test in patients with symptomatic knee osteoarthritis. *Rheumatol Int*. 2013;33(10):2691-2694. doi:10.1007/s00296-012-2512-3

17. Alghadir A, Anwer S, Brismée JM. The reliability and minimal detectable change of Timed Up and Go test in individuals with grade 1-3 knee osteoarthritis. *BMC Musculoskelet Disord*. 2015;16:174. doi:10.1186/s12891-015-0637-8

18. Jonsson E, Seiger A, Hirschfeld H. One-leg stance in healthy young and elderly adults: a measure of postural steadiness? *Clin Biomech (Bristol, Avon)*. 2004;19(7):688-694. doi:10.1016/j.clinbiomech.2004.04.002

19. Walk D, Sehgal N, Moeller-Bertram T, et al. Quantitative Sensory Testing and

Mapping: A Review of Nonautomated Quantitative Methods for Examination of the Patient With Neuropathic Pain. *The Clinical Journal of Pain*. 2009;25(7):632. doi:10.1097/AJP.0b013e3181a68c64

20. Keizer D, van Wijhe M, Post WJ, Wierda JMKH. Quantifying Allodynia in Patients Suffering From Unilateral Neuropathic Pain Using Von Frey Monofilaments. *The Clinical Journal of Pain*. 2007;23(1):85. doi:10.1097/01.ajp.0000210950.01503.72

21. Rolke R, Baron R, Maier C, et al. Quantitative sensory testing in the German Research Network on Neuropathic Pain (DFNS): standardized protocol and reference values. *Pain*. 2006;123(3):231-243. doi:10.1016/j.pain.2006.01.041

Appendix A1. Accuracy statistics with 95% confidence intervals for the individual and initially potential predictor variables.

Variables	Cutoff	Sensitivity at cut point	95% CI LB	95% CI UB	Specificity at cut point	95% CI LB	95% CI UB	AUC at cut point	Positive Likelihood	95% CI LB	95% CI UB
**Pain Related Variables**											
BPI Interference - Pain Impact on Function *	≤ 2.79	0.47	0.46	0.48	0.83	0.80	0.86	0.65	2.76	2.74	2.79
VAS Pain NOW	≤ 3.70	0.87	0.84	0.90	0.42	0.40	0.44	0.64	1.50	1.47	1.53
VAS Pain WEEK	≤ 7.10	0.93	0.89	0.97	0.42	0.40	0.44	0.68	1.60	1.58	1.63
**Cognitive Status Variables**											
Mini-Mental	≤ 23.5	0.87	0.84	0.90	0.39	0.37	0.41	0.63	1.43	1.40	1.45
**Psychosocial-Related Variables**											
BECK Depression Inventory	≤ 10.5	0.67	0.65	0.69	0.58	0.56	0.60	0.62	1.60	1.58	1.61
VAMS Sleepiness	≤ 2.9	0.67	0.65	0.69	0.39	0.37	0.41	0.53	1.10	1.08	1.12
**Self-Reported Health-Related Quality of Life**											
SF-12 Physical Component Score	≤ 37.87	0.87	0.84	0.90	0.31	0.29	0.33	0.59	1.26	1.23	1.29
SF-12 Mental Component Score	≤ 52.59	0.67	0.65	0.69	0.56	0.55	0.57	0.61	1.52	1.50	1.54
**Disease Specific Measures**											
Lequesne Max-Distance Walked Score	≤ 4.50	0.53	0.52	0.54	0.39	0.37	0.41	0.46	0.87	0.85	0.88
Womac Function Score	≤ 31	0.53	0.52	0.54	0.75	0.72	0.78	0.64	2.12	2.10	2.14
Womac TOTAL Score	≤ 43.5	0.60	0.58	0.62	0.72	0.69	0.75	0.66	2.14	2.12	2.16
**Quantitative Sensory Testing Variables**											
Von-Frey Sensation - HAND	≤ 0.50	0.27	0.24	0.30	0.50	0.50	0.50	0.38	0.54	0.52	0.56
Von-Frey Pin-Prick - HAND	≤ 5	0.87	0.84	0.90	0.42	0.40	0.44	0.64	1.50	1.47	1.53
Von-Frey Pain Threshold - HAND	≤ 240	0.73	0.70	0.76	0.47	0.46	0.48	0.60	1.38	1.36	1.40
Pain Pressure Threshold PRE CPM - HAND	≤ 2.84	0.53	0.52	0.54	0.75	0.72	0.78	0.64	2.12	2.10	2.14
Change in PPT after CPM - HAND	≤ 6.38	0.33	0.31	0.35	0.44	0.43	0.45	0.39	0.59	0.57	0.61
Change in PPT after CPM - KNEE	≤ 21.29	0.60	0.58	0.62	0.61	0.59	0.63	0.61	1.54	1.52	1.56

n= 51 cases present for all variables

TRIPOD Checklist: Prediction Model Development

Section/Topic	Item	Checklist Item	Page
**Title and abstract**			
Title	1	Identify the study as developing and/or validating a multivariable prediction model, the target population, and the outcome to be predicted.	5-6
Abstract	2	Provide a summary of objectives, study design, setting, participants, sample size, predictors, outcome, statistical analysis, results, and conclusions.	2
**Introduction**			
Background and objectives	3a	Explain the medical context (including whether diagnostic or prognostic) and rationale for developing or validating the multivariable prediction model, including references to existing models.	3-5
	3b	Specify the objectives, including whether the study describes the development or validation of the model or both.	5-6
**Methods**			
Source of data	4a	Describe the study design or source of data (e.g., randomized trial, cohort, or registry data), separately for the development and validation data sets, if applicable.	5 & supplemental material
	4b	Specify the key study dates, including start of accrual; end of accrual; and, if applicable, end of follow-up.	5
Participants	5a	Specify key elements of the study setting (e.g., primary care, secondary care, general population) including number and location of centres.	supplemental material
	5b	Describe eligibility criteria for participants.	supplemental material
	5c	Give details of treatments received, if relevant.	supplemental material
Outcome	6a	Clearly define the outcome that is predicted by the prediction model, including how and when assessed.	5-6
	6b	Report any actions to blind assessment of the outcome to be predicted.	Reference 72
Predictors	7a	Clearly define all predictors used in developing or validating the multivariable prediction model, including how and when they were measured.	supplemental m. & Tables 1 Appendix 1
	7b	Report any actions to blind assessment of predictors for the outcome and other predictors.	N/A
Sample size	8	Explain how the study size was arrived at.	Reference 72
Missing data	9	Describe how missing data were handled (e.g., complete-case analysis, single imputation, multiple imputation) with details of any imputation method.	N/A
Statistical analysis methods	10a	Describe how predictors were handled in the analyses.	6-7
	10b	Specify type of model, all model-building procedures (including any predictor selection), and method for internal validation.	6-7
	10d	Specify all measures used to assess model performance and, if relevant, to compare multiple models.	6/7
Risk groups	11	Provide details on how risk groups were created, if done.	N/A
**Results**			
Participants	13a	Describe the flow of participants through the study, including the number of participants with and without the outcome and, if applicable, a summary of the follow-up time. A diagram may be helpful.	N/A
	13b	Describe the characteristics of the participants (basic demographics, clinical features, available predictors), including the number of participants with missing data for predictors and outcome.	Table 1 and Reference 34
Model development	14a	Specify the number of participants and outcome events in each analysis.	8
	14b	If done, report the unadjusted association between each candidate predictor and outcome.	N/A
Model specification	15a	Present the full prediction model to allow predictions for individuals (i.e., all regression coefficients, and model intercept or baseline survival at a given time point).	8 & table 2
	15b	Explain how to the use the prediction model.	8-12
Model performance	16	Report performance measures (with CIs) for the prediction model.	Table 3
**Discussion**			
Limitations	18	Discuss any limitations of the study (such as nonrepresentative sample, few events per predictor, missing data).	12
Interpretation	19b	Give an overall interpretation of the results, considering objectives, limitations, and results from similar studies, and other relevant evidence.	9-12
Implications	20	Discuss the potential clinical use of the model and implications for future research.	9-12
**Other information**			
Supplementary information	21	Provide information about the availability of supplementary resources, such as study protocol, Web calculator, and data sets.	Reference 72 & 34
Funding	22	Give the source of funding and the role of the funders for the present study.	Title Page

We recommend using the TRIPOD Checklist in conjunction with the TRIPOD Explanation and Elaboration document.
